# Characterizing the epidemiology and interaction between HIV-1 and HBV co-infection in South Africa

**DOI:** 10.1186/1471-2334-14-S2-P18

**Published:** 2014-05-23

**Authors:** Philippa C Matthews, Apostolos Beloukas, Amna Malik, Thumbi Ndung’u, Philip Goulder, Anna Maria Geretti, Paul Klenerman

**Affiliations:** 1 Nuffield Department of Medicine, University of Oxford, Oxford OX1 3SY, UK; 2 Department of Infectious Diseases and Microbiology, Oxford University Hospitals, John Radcliffe Hospital, Headington, Oxford OX3 9DU, UK; 3 Institute of Infection & Global Health, University of Liverpool, Liverpool L69 7BE, UK; 4 Department of Pediatrics, University of Oxford, Peter Medawar Building, Oxford OX1 3SY, UK; 5 HIV Pathogenesis Program, Doris Duke Medical Research Institute, University of KwaZulu-Natal, Durban, South Africa; 6 NIHR Biomedical Research Center, John Radcliffe Hospital, Headington, Oxford OX3 9DU, UK

## Introduction

Anti-Retroviral Therapy (ART) has dramatically reduced morbidity and mortality associated with HIV/AIDS. However, this has left a niche for the emergence of liver disease in HIV-positive individuals co-infected with HBV. Despite the geographical overlap between highly endemic HBV and HIV in Southern Africa, there is a wide range in the prevalence of co-infection. We therefore set out to characterize the epidemiology of HIV/HBV co-infection in a Durban cohort, and to investigate the possible impact of HBV infection on HIV disease progression.

## Materials and methods

We investigated a cohort of 498 adult women recruited via antenatal/postnatal clinics in Durban, South Africa, of whom 72 were HIV negative and 426 were chronically HIV-infected and ART-naïve (median CD4 count 368 cells/mm3, median HIV-1 RNA load 4.47 log10 copies/ml). We screened plasma for HBsAg by ELISA (Biokit). CD8+ T cell responses to HIV peptides were quantified by IFN-gamma ELISpot assay in 325 HIV-infected individuals including 35 with HBV coinfection.

## Results

Overall HBsAg prevalence was 46/498 (9.2%; 95%-confidence interval 7-12%); coinfection rates were 9.4% in HIV-positive and 8.3% in HIV-negative individuals. CD4 counts were significantly lower in with HBV/HIV coinfection than with HIV monoinfection (302 vs. 375 cells/mm3; p=0.02). However, HBV status made no significant impact on HIV viral load (4.49 log10 copies/ml in coinfection vs. 4.46 log10 in monoinfection). There was no difference in breadth, magnitude, or protein-specificity of IFN-gamma responses to HIV according to HBV status.

## Conclusions

In this cohort of Durban women, 9% were coinfected with HBV. Women with HIV/HBV co-infection had significantly lower CD4 counts, highlighting the potential detriment of coinfection. However, in a small subset we did not find a difference in CD8+ T cell responses to HIV. These data contribute towards an improved understanding of the scale of the HIV/HBV coinfection problem in Africa, and suggest that adverse outcomes are mediated by factors other than CD8+ T cell responses to HIV.

